# Pregnancy Outcomes After Belatacept Exposure in Solid Organ Transplant Recipients: A Scoping Review

**DOI:** 10.3390/healthcare14162561

**Published:** 2026-08-16

**Authors:** Ibrahim Tawhari, Manal Alotaibi, Hany El Hennawy, Fatmah Yamani, Muath Alqahtani, Khalid Asiri, Mohammed Tawhari

**Affiliations:** 1 Department of Internal Medicine, Section of Nephrology, College of Medicine, King Khalid University, Abha 61421, Saudi Arabia; 2 Department of Internal Medicine, Section of Nephrology, College of Medicine, Umm Al-Qura University, Makkah 21955, Saudi Arabia; meotaibi@uqu.edu.sa; 3 Surgery Department, Section of Transplantation, King Abdulaziz Medical City, Ministry of National Guard-Health Affairs, Jeddah 21423, Saudi Arabia; hennawyhany2@hotmail.com; 4 King Abdullah International Medical Research Center, Jeddah 21423, Saudi Arabia; 5 Department of Nephrology, King Faisal Specialist Hospital and Research Center, Jeddah 21499, Saudi Arabia; f.n.yamani@gmail.com; 6 College of Medicine, King Khalid University, Abha 61421, Saudi Arabia; muath.a.alqahtani@gmail.com; 7 Department of Nephrology, Armed Forces Hospitals Southern Region, Khamis Mushayte 61961, Saudi Arabia; dr-khalid2030@hotmail.com; 8 College of Medicine, King Saud Bin Abdulaziz University for Health Sciences, Riyadh 11481, Saudi Arabia; tawharim@ksau-hs.edu.sa; 9 Department of Medicine, Division of Nephrology, King Abdulaziz Medical City, Ministry of the National Guard-Health Affairs, Riyadh 11426, Saudi Arabia; 10 King Abdullah International Medical Research Center, Riyadh 11481, Saudi Arabia

**Keywords:** belatacept, pregnancy, kidney transplantation, solid organ transplantation, immunosuppression, maternal outcomes, fetal outcomes, calcineurin inhibitor-sparing, biologic immunosuppression, maternal–fetal medicine

## Abstract

**Highlights:**

**What are the main findings?**

- No congenital malformations were reported among the 16 live-born infants exposed to belatacept in utero; however, this number is far too small to characterize teratogenic risk.
- Preeclampsia was reported in 8 of 16 pregnancies in the largest case series; low birth weight (<2500 g) was reported in all live-born infants, largely in the context of preterm delivery.

**What are the implications of the main findings?**

- Stable allograft function was reported in 14 of 19 pregnancies with follow-up data, with no rejection episodes during belatacept exposure, suggesting continued immunosuppressive efficacy.
- Prospective multicenter registries are needed to establish the safety of belatacept during pregnancy.

**Abstract:**

**Background**: Pregnancy after solid organ transplantation carries increased maternal and fetal risks, compounded by the teratogenicity, nephrotoxicity, and metabolic effects of available immunosuppressive agents. Belatacept, a calcineurin inhibitor-sparing T-cell costimulation blocker with favorable renal and metabolic profiles, has emerged as an alternative; however, evidence regarding its safety during pregnancy remains scarce. **Methods**: A scoping review was conducted per PRISMA-ScR recommendations. PubMed, Google Scholar, Web of Science, Scopus, and the Cochrane Library were searched (January 2015–March 2025), supplemented by citation searching, for studies reporting pregnancy outcomes in solid organ transplant recipients receiving belatacept. Methodological quality was assessed using Joanna Briggs Institute critical appraisal tools. **Results**: Three studies (one case series and two case reports) encompassing 21 pregnancies among 15 recipients were included, predominantly in kidney transplant recipients; several recipients contributed more than one pregnancy, so pregnancy-level outcomes are not statistically independent. Sixteen pregnancies resulted in live birth and five ended in miscarriage, at least four of which occurred in pregnancies with periconception mycophenolate exposure. No congenital malformations were reported among live-born infants, although the number of exposures is far too small to characterize teratogenic risk. Stable allograft function was reported in 14 of 19 pregnancies with available follow-up, with no rejection episodes during belatacept exposure. Maternal complications included preeclampsia (8 of 16 in the case series), gestational diabetes, cytomegalovirus reactivation, and acute kidney injury. Low birth weight (<2500 g) was reported in all live births, predominantly in the context of preterm delivery. **Conclusions**: The published cases have not identified congenital malformations to date, but the number of documented exposures is far too small to characterize teratogenic risk, and the overall certainty of evidence is very low. Because no comparative studies were available, maternal and neonatal outcomes cannot be assumed equivalent to those of conventional immunosuppression. Belatacept cannot be recommended for routine use during pregnancy; clinical decisions must remain individualized, and preconception counseling and prospective multicenter registries are urgently needed.

## 1. Introduction

Advances in transplantation and long-term immunosuppressive management have substantially improved survival among women of reproductive age living with solid organ allografts. As graft longevity and quality of life continue to improve, pregnancy after transplantation is becoming increasingly common, particularly among kidney transplant recipients. Despite these advances, pregnancy in transplant recipients remains medically complex and carries substantially higher risks than pregnancy in the general population. Maternal hypertension, preeclampsia, gestational diabetes, fetal growth restriction, preterm birth, and allograft dysfunction occur at disproportionately elevated rates, even under optimal multidisciplinary care [1,2,3,4].

A major challenge in this setting lies in the limitations of currently available immunosuppressive strategies. Several standard agents used to preserve allograft function are associated with important maternal or fetal toxicities. Mycophenolate mofetil is strongly associated with first-trimester pregnancy loss and congenital malformations involving the craniofacial, cardiac, and auditory systems [5]. Consequently, women receiving mycophenolate are advised to discontinue treatment before conception whenever feasible. Calcineurin inhibitors, particularly tacrolimus and cyclosporine, are generally considered compatible with pregnancy and remain the backbone of maintenance immunosuppression in most transplant programs. Nevertheless, these agents are frequently complicated by nephrotoxicity, endothelial dysfunction, hypertension, neurotoxicity, and metabolic disturbances that may worsen maternal and obstetric outcomes [6,7]. Belatacept utilization has progressively increased worldwide, particularly in kidney transplantation, driven by its favorable renal and metabolic profile compared with calcineurin inhibitors.

Within this evolving therapeutic landscape, belatacept has generated increasing interest as a calcineurin inhibitor-sparing strategy. Belatacept is a selective T-cell costimulation blocker composed of a cytotoxic T-lymphocyte-associated antigen 4 immunoglobulin fusion protein that inhibits the CD28-CD80/86 signaling pathway and prevents T-cell activation [8,9]. In kidney transplantation, belatacept-based maintenance regimens have demonstrated favorable renal hemodynamic profiles and improved long-term kidney function compared with calcineurin inhibitor-based therapy in selected populations [10]. Unlike calcineurin inhibitors, belatacept does not induce afferent arteriolar vasoconstriction and is not intrinsically nephrotoxic, features that may hold particular relevance during pregnancy when preservation of maternal renal function is critical.

Theoretical advantages of belatacept in pregnancy extend beyond nephrotoxicity avoidance. Experimental data suggest minimal placental transfer during early gestation and no clear evidence of direct teratogenicity in animal studies. In contrast to antiproliferative agents such as mycophenolate, belatacept lacks direct cytotoxic effects on rapidly dividing fetal tissues. These mechanistic considerations have prompted interest in whether belatacept might offer a safer immunologic platform for selected women contemplating pregnancy, particularly those unable to tolerate calcineurin inhibitors or requiring withdrawal of teratogenic agents before conception.

Clinical evidence, however, remains remarkably sparse. Pregnant transplant recipients are rarely included in prospective immunosuppressive studies, and most available information regarding belatacept exposure derives from isolated case reports and small observational series. Notably, most transplant pregnancy registries were developed during the calcineurin-inhibitor era and lack structured data collection for newer biologic agents such as belatacept. Consequently, clinicians are often forced to make difficult management decisions in the absence of reliable safety data. The uncertainty extends not only to fetal outcomes, but also to maternal complications, graft stability, breastfeeding safety, and long-term pediatric development after in utero exposure.

This scoping review was therefore conducted to provide a focused synthesis of the currently available literature. Specifically, we sought to evaluate reported maternal, fetal, neonatal, and graft-related outcomes; examine patterns of immunosuppressive management surrounding conception and pregnancy; compare the observed outcomes with established transplant pregnancy literature; and identify critical gaps that continue to limit evidence-based clinical decision-making in this emerging area of transplant medicine.

## 2. Materials and Methods

This scoping review was conducted and reported in accordance with the Preferred Reporting Items for Systematic Reviews and Meta-Analyses Extension for Scoping Reviews (PRISMA-ScR) guidelines [11]. The completed PRISMA-ScR checklist is provided as Supplementary Table S1. The review protocol was retrospectively registered in the Open Science Framework (OSF; registration DOI: 10.17605/OSF.IO/G8JPF). Because of the anticipated descriptive nature and heterogeneity of eligible studies, meta-analysis was not feasible; this review therefore provides a narrative synthesis of available evidence. We acknowledge that the protocol was registered retrospectively, which reduces methodological transparency; this limitation is noted below. We deliberately adopted a scoping-review framework rather than a systematic review with meta-analysis, because our objective was to map the extent, nature, and distribution of the available evidence rather than to derive pooled effect estimates; accordingly, the aggregate proportions reported throughout are descriptive only and should not be interpreted as quantitative evidence synthesis.

A systematic literature search was performed using PubMed, Google Scholar, Web of Science, Scopus, and the Cochrane Library to identify studies published between January 2015 and March 2025. Search terms included combinations of “belatacept,” “CTLA-4-Ig,” “pregnancy,” “pregnant women,” “gestation,” “solid organ transplant,” “solid organ transplantation,” “kidney transplant,” and “liver transplant.” The full PubMed search strategy, which could be adapted for all databases, was: (belatacept[tiab] OR “CTLA-4-Ig”[tiab] OR LEA29Y[tiab]) AND (pregnan*[tiab] OR gestation*[tiab] OR “pregnant women”[tiab]) AND (“solid organ transplant*”[tiab] OR “kidney transplant*”[tiab] OR “renal transplant*”[tiab] OR “liver transplant*”[tiab] OR “heart transplant*”[tiab] OR “lung transplant*”[tiab]); filters applied: publication date 2015/01/01–2025/03/31. No language restrictions were initially applied during the search process; however, only English-language studies were ultimately included in the final review because of the absence of eligible non-English reports meeting inclusion criteria.

**Figure 1:**
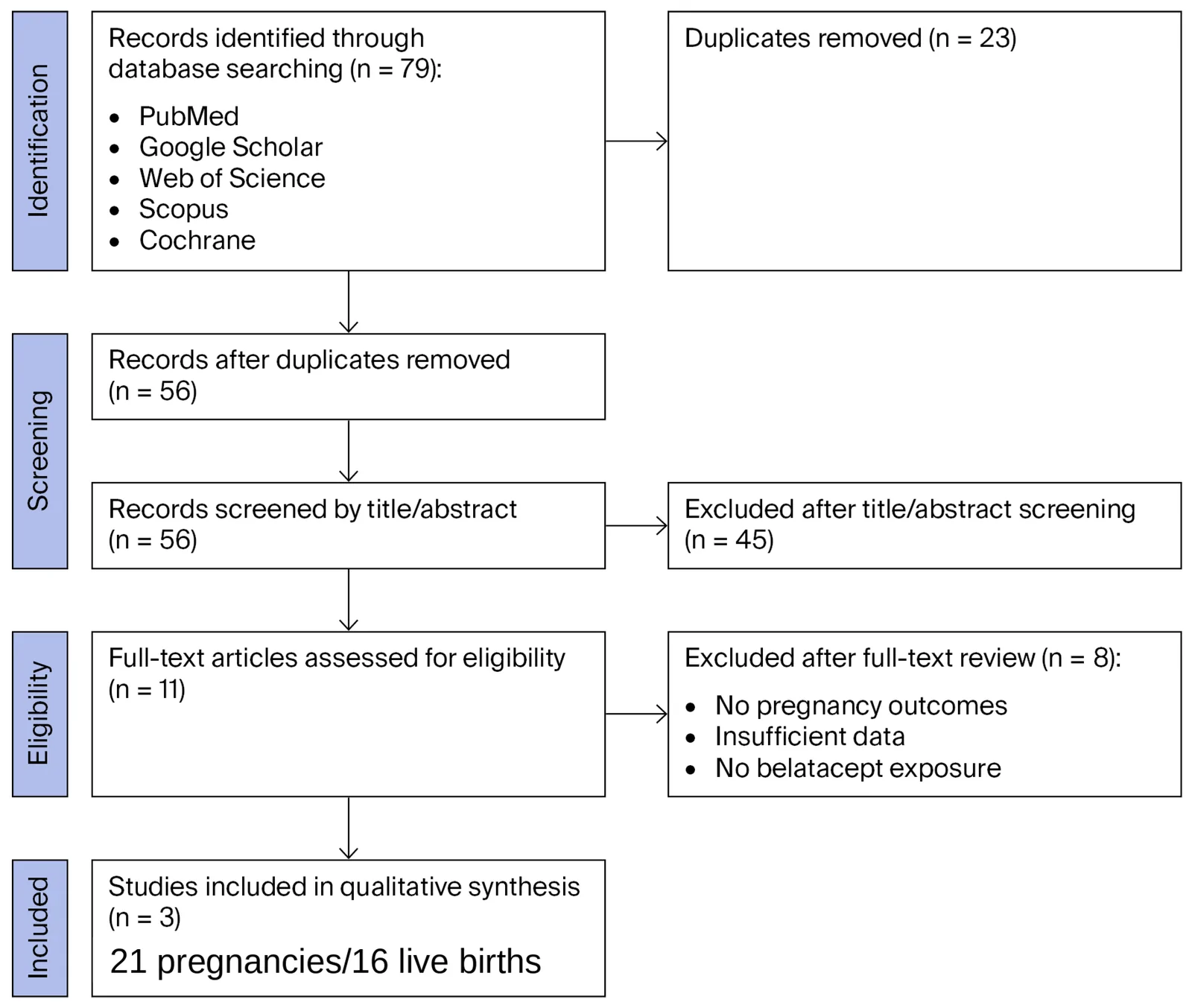
PRISMA-ScR flow diagram of study identification, screening, eligibility, and inclusion.

The number of records retrieved from each database (PubMed, Scopus, Web of Science, the Cochrane Library, and Google Scholar) was recorded and is reported in the PRISMA-ScR flow diagram (Figure 1). In addition to database searching, the reference lists of all included studies and relevant review articles were screened manually (backward citation searching) to identify further eligible reports. Google Scholar was searched as a supplementary source to capture reports not indexed in the primary databases; because of its lower reproducibility, it was used only to identify additional candidate records, each of which was subsequently verified in an indexed database. The search was restricted to January 2015 onward because, although belatacept received U.S. FDA approval in June 2011, it entered broader clinical use for maintenance immunosuppression only in subsequent years, and the earliest published pregnancy exposures date from 2015; screening of records without a start-date restriction identified no eligible pregnancy reports before 2015. Because several of the included publications shared authors and drew in part on the Transplant Pregnancy Registry International, we specifically checked for overlapping pregnancies between reports; the case series (Georgia) and the two case reports (Michigan and Texas) described distinct recipients, and no duplicate pregnancies were identified. The authors of the primary studies were not formally contacted for additional individual-level data, which is acknowledged as a limitation.

After the search was completed, duplicate records were removed manually using a standard citation manager (Zotero 9.0.1). The remaining unique records underwent title and abstract screening.

Eligible studies included case reports, case series, cohort studies, and observational studies describing pregnancy outcomes among solid organ transplant recipients receiving belatacept as part of maintenance immunosuppressive therapy. Kidney, liver, heart, lung, and combined organ transplant recipients were considered eligible. Studies were required to report at least one pregnancy-related maternal, fetal, neonatal, or graft outcome. Reviews, editorials, conference abstracts without sufficient clinical detail, animal studies, protocols, and grey literature were excluded (Table 1).

Two reviewers independently screened titles, abstracts, and full-text articles for eligibility. Disagreements were resolved through discussion and consensus, with involvement of a third reviewer when necessary.

Data extraction was performed using a standardized collection form. Extracted variables included study design, country, recipient demographics, transplant characteristics, interval between transplantation and pregnancy, belatacept regimen, concomitant immunosuppressive therapy, maternal complications, graft outcomes, gestational age at delivery, birth weight, congenital anomalies, neonatal complications, and breastfeeding outcomes. Given the descriptive nature and heterogeneity of the included studies, findings were synthesized narratively. Aggregate proportions were presented descriptively to summarize reported outcomes and were not intended for inferential statistical comparison.

Methodological quality was assessed using the Joanna Briggs Institute critical appraisal checklists for case reports and case series. Although all included studies satisfied at least 70% of appraisal criteria, interpretation of findings remained limited by small sample size, lack of comparator groups, incomplete follow-up, and substantial risk of publication bias. This review is based exclusively on previously published literature; compliance with the Declaration of Helsinki and the Declaration of Istanbul regarding organ donor source is assumed for all included primary studies. The search was confined to peer-reviewed publications; transplant pregnancy and pharmacovigilance registries (e.g., the Transplant Pregnancy Registry International), regulatory adverse-event databases (e.g., the FDA Adverse Event Reporting System), conference abstracts, dissertations, and other grey-literature sources were not systematically searched. As discussed further among the limitations, this decision improved the consistency and feasibility of critical appraisal but may have excluded relevant unpublished exposures and contributed to publication bias.

## 3. Results

### 3.1. Study Selection

The initial database search identified 79 records. After removal of duplicates and preliminary screening, 56 unique studies underwent title and abstract review. Forty-five articles were excluded because they did not satisfy inclusion criteria. Full-text assessment was subsequently performed for 11 studies. Eight studies were excluded owing to lack of pregnancy-specific clinical outcomes, insufficient data availability, or failure to evaluate belatacept exposure during pregnancy. Ultimately, three studies were included in the final synthesis: one case series [12] and two case reports, one of which described two separate patient narratives [13,14]. The study selection process is illustrated in the PRISMA flow diagram (Figure 1).

### 3.2. Study Characteristics

The available evidence consisted exclusively of low-level observational data. The largest study was a case series by Coscia et al. [12], which described 16 pregnancies among 12 kidney transplant recipients. Two additional reports contributed five pregnancies involving three recipients [13,14]. In total, 21 pregnancies among 15 recipients were identified across all included studies.

Most pregnancies occurred in kidney transplant recipients, while one case involved a liver transplant recipient [14]. Several women contributed more than one pregnancy, and therefore pregnancies should not be interpreted as statistically independent events. All studies originated from transplant centers in the United States, specifically Georgia, Michigan, and Texas.

Recipient age ranged from 28 to 37 years. The mean interval between transplantation and pregnancy was approximately four years, with reported ranges extending from 1.25 to 8 years. Most recipients had undergone living donor transplantation, although one liver recipient had previously received both living donor and deceased donor grafts [14] (Table 2).

**Figure 2:**
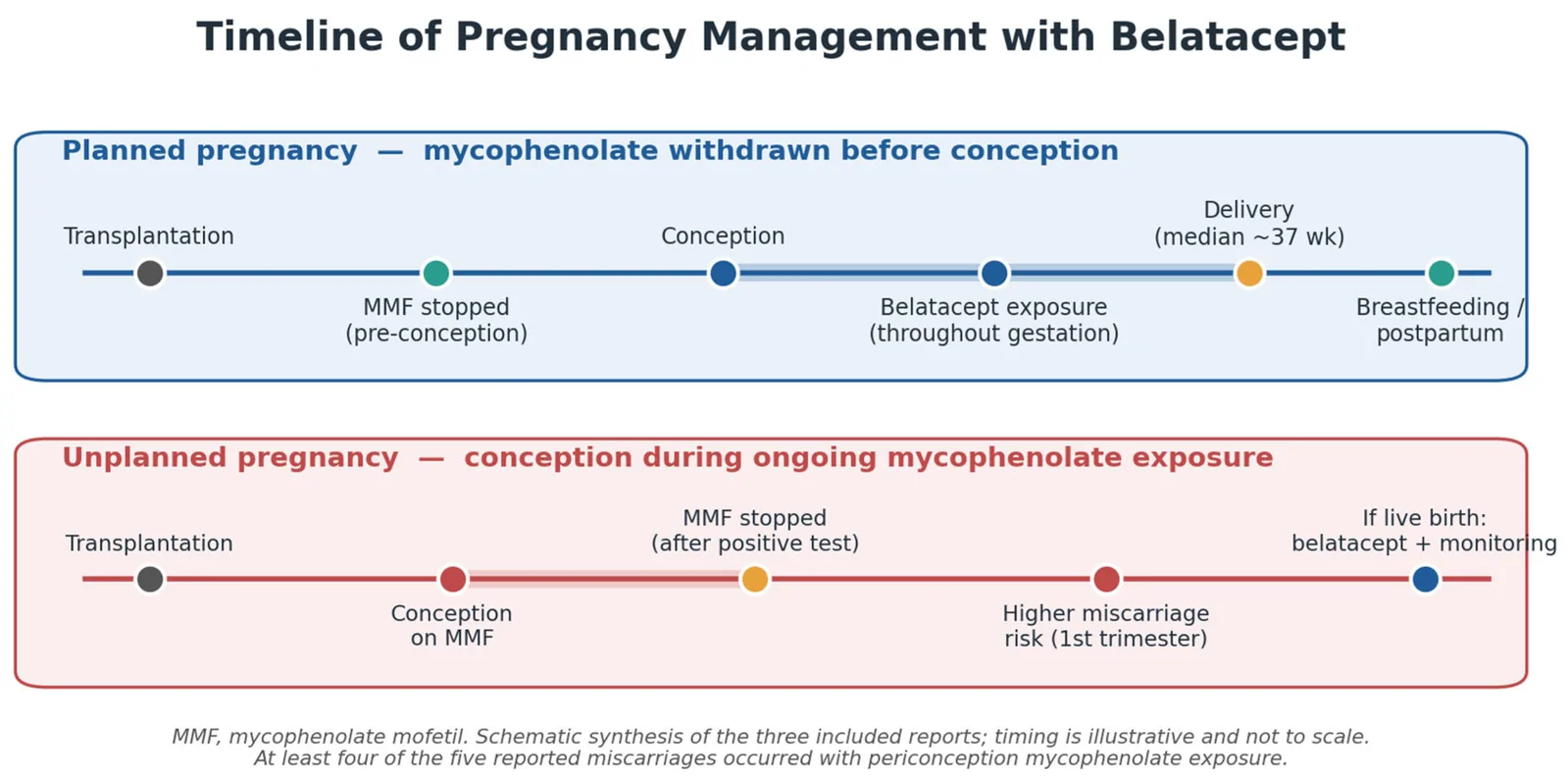
Schematic timeline of pregnancy management with belatacept, contrasting planned pregnancies (mycophenolate withdrawn before conception) with unplanned pregnancies (conception during ongoing mycophenolate exposure).

Belatacept was administered using standard maintenance intravenous dosing protocols, generally consisting of 5 mg/kg or 10 mg/kg monthly infusions. None of the included studies reported dose modification during pregnancy or therapeutic drug monitoring. Several pregnancies followed planned discontinuation of mycophenolate before conception, whereas others resulted from unplanned conception during ongoing mycophenolate exposure [12,13]. A schematic timeline of pregnancy management with belatacept across the included reports is provided in Figure 2.

### 3.3. Pregnancy and Fetal Outcomes

Among the 21 reported pregnancies, 16 resulted in live birth and 5 ended in miscarriage (16 of 21 and 5 of 21, respectively). Importantly, at least four of the five miscarriages occurred in pregnancies with periconception mycophenolate exposure—two of the three miscarriages in the case series and both miscarriages in the multiparous case-report recipient, who conceived during ongoing mycophenolate therapy [12,13]. Because mycophenolate is an established cause of first-trimester pregnancy loss, the aggregate miscarriage proportion cannot be attributed to belatacept; outcomes are therefore presented separately according to mycophenolate exposure and pregnancy planning in Table 3 and in the patient-level Appendix A (Table A1).

In the largest series, the median gestational age among live births was 37 weeks (range 34.8–37.9) [12], and preterm delivery was common, consistent with the broader transplant-pregnancy literature. Low birth weight, defined as a birth weight below 2500 g, was reported in all live-born infants. However, individual birth weights and gestational-age–adjusted (small-for-gestational-age) classifications were not reported in the source studies; low birth weight in this cohort therefore appears to reflect preterm delivery rather than confirmed fetal growth restriction, and preterm birth, low birth weight, and small-for-gestational-age status could not be reliably distinguished. Neonatal follow-up was limited: one neonate required neonatal intensive-care-unit observation, no major neonatal complications were described, and no structured neonatal follow-up beyond the perinatal period was reported [12].

No congenital anomalies or structural malformations were reported among any of the 16 live-born infants. The published cases have thus not identified congenital malformations to date; however, the number of documented exposures is far too small to characterize teratogenic risk, and neither a modest increase in common malformations nor rare but important congenital effects can be excluded. Detailed outcome data are presented in Table 4, and patient-level data are provided in Table A1.

### 3.4. Maternal Complications

Maternal complications were frequent, especially in the case series. Preeclampsia was reported in 8 of 16 pregnancies in the series [12]. Additional maternal complications across the three studies included gestational diabetes mellitus (1 of 16), cytomegalovirus reactivation (1 of 16), COVID-19 infection (2 of 16), and worsening proteinuria or acute kidney injury (1 of 16) [12]. Among the case reports, one recipient developed preeclampsia but had stable renal function postpartum [13], whereas the liver transplant recipient had no maternal complications [14].

### 3.5. Breastfeeding Outcomes

Data on breastfeeding were limited and are summarized relative to the number of mothers with a live birth rather than to all pregnancies. In the case series, 7 of 13 mothers with a live birth breastfed their infants [12]. One case report described successful breastfeeding for 12 months without any reported adverse effect on the infant [14]. No breastfeeding data were reported by Combs et al. [13].

### 3.6. Allograft Outcome Summary

For the purposes of this review, stable or adequate graft function was defined—on the basis of the variables available in the source reports—as the absence of biopsy-proven rejection, graft loss, or a sustained rise in serum creatinine or new-onset significant proteinuria during the reported follow-up period. By this definition, stable graft function was maintained in 14 of 19 pregnancies with reported follow-up, and no rejection episodes occurred during belatacept-exposed pregnancies in the case series [12]. One case-report recipient experienced acute cellular and antibody-mediated rejection after a first-trimester miscarriage while receiving mycophenolate mofetil, and subsequently lost graft function six months postpartum in the context of nonadherence to scheduled belatacept infusions [13]. The single liver transplant recipient maintained stable graft function throughout pregnancy and the 12-month postpartum period [14]. The source reports varied in the availability of serum creatinine, proteinuria, biopsy, rejection, graft-survival, and follow-up-duration data; a single aggregate proportion of “stable graft function” should therefore be interpreted with caution, and available patient-level detail is provided in Table A1.

### 3.7. Concomitant Immunosuppressive Regimens

The immunosuppressive regimens at the time of conception or during pregnancy varied across the included studies. Table 4 summarizes the concomitant medications and the timing of mycophenolate discontinuation where applicable.

## 4. Discussion

This is the first focused synthesis of pregnancy outcomes following belatacept exposure across all solid organ transplant types. The review consolidates the currently available clinical evidence, which remains limited to 21 reported pregnancies, predominantly in kidney transplant recipients. Although the data are sparse, several observations are clinically relevant for transplant physicians managing women of reproductive age.

Perhaps the most notable observation is the absence of reported congenital anomalies among the 16 live-born infants exposed to belatacept during gestation. This observation does not establish fetal safety: the published cases have not identified congenital malformations to date, but the number of exposures is far too small to characterize teratogenic risk. With only 16 live births, the available evidence cannot exclude either a modest increase in common malformations or rare but important congenital effects, and hundreds of prospectively followed pregnancies would likely be required before clinically meaningful reassurance could be established.

The maternal and neonatal outcomes observed in these reports cannot be formally compared with those of conventional immunosuppression, because no comparative studies were included and the belatacept-exposed recipients had complex transplant histories—including prior calcineurin-inhibitor toxicity, previous rejection, impaired graft function, and concomitant mycophenolate exposure. Although contemporary registry data from kidney transplant recipients on standard maintenance immunosuppression describe live-birth proportions of approximately 75–85%, together with high rates of preeclampsia, prematurity, and fetal growth restriction [4], differences in baseline risk, transplant era, graft type, clinical selection, pregnancy planning, and outcome definitions preclude meaningful comparison. Descriptive similarity to historical estimates should not be interpreted as evidence of equivalent safety.

Additional maternal complications across the three studies included gestational diabetes (1 of 16), cytomegalovirus reactivation (1 of 16), COVID-19 infection (2 of 16), and worsening proteinuria or acute kidney injury (1 of 16) [12]. None of these complications is mechanistically linked to belatacept’s selective T-cell costimulation blockade, and their distribution is broadly consistent with the immunocompromised state inherent to transplantation. The small number of reported pregnancies precludes any meaningful incidence comparison with calcineurin inhibitor-based regimens.

At the same time, the available reports describe several clinical scenarios in which belatacept may hold particular relevance. Many recipients receiving belatacept had prior calcineurin-inhibitor toxicity, difficult hypertension, a rejection history, or concern regarding preservation of long-term renal function [12,13,14]. Unlike calcineurin inhibitors, belatacept does not induce chronic renal vasoconstriction or cumulative nephrotoxicity, and preservation of maternal renal reserve may in theory be advantageous in recipients with reduced baseline graft function, vascular disease, or chronic hypertension. These considerations are, however, theoretical and hypothesis-generating rather than evidence-based: preservation of maternal renal reserve and any reduction in hypertension risk with belatacept during pregnancy have not been demonstrated in the available data and should be regarded as biologically plausible hypotheses only.

Available pregnancy experience with other biologic agents in transplantation, such as rituximab and eculizumab, remains similarly limited, although belatacept’s IgG1 Fc structure theoretically minimizes placental transfer during the first trimester relative to smaller molecules [15,16]. This mechanistic distinction may partly explain the absence of early teratogenic signals. A comparative overview of pregnancy-relevant considerations across common immunosuppressive agents is provided in Table 5.

The relationship between pregnancy planning and outcome also emerged repeatedly throughout the reviewed literature. Unplanned pregnancies occurring during ongoing mycophenolate exposure were associated with miscarriage and rejection-related complications [13]. In contrast, pregnancies preceded by planned withdrawal of teratogenic agents and careful multidisciplinary monitoring appeared to achieve more favorable maternal and neonatal outcomes. This distinction underscores the importance of structured reproductive counseling within transplant programs, particularly for younger women receiving complex immunosuppressive regimens.

Belatacept’s immunologic mechanism further distinguishes it from traditional maintenance agents. By selectively blocking CD28-CD80/86-mediated T-cell costimulation, belatacept avoids the antiproliferative and direct cytotoxic effects associated with mycophenolate exposure. Experimental models have not demonstrated clear teratogenicity, although translation of animal findings to human pregnancy remains inherently limited. Under the current FDA Pregnancy and Lactation Labeling Rule (PLLR), belatacept’s prescribing information advises use during pregnancy only if clearly needed, reflecting the absence of adequate controlled human data.

Importantly, belatacept use is contraindicated in Epstein–Barr virus-seronegative recipients because of the increased risk of post-transplant lymphoproliferative disorder [17]. This consideration is particularly relevant in women of reproductive age contemplating long-term therapy.

### 4.1. Placental Transfer and Trimester-Specific Exposure

As an IgG1 fusion protein, belatacept is expected to cross the human placenta primarily during the second and third trimesters via neonatal Fc receptor-mediated transport, potentially leading to fetal exposure later in gestation [15,16]. First-trimester transfer is minimal, which may explain the absence of early teratogenic signals. Currently, no included study provides trimester-specific data on belatacept exposure, limiting any assessment of differential risks based on gestational timing. Future pharmacokinetic studies in pregnancy should aim to characterize maternal and cord blood concentrations at delivery.

### 4.2. Ethical Considerations

Ethical considerations are paramount. Pregnant transplant recipients must balance their desire for motherhood against uncertain fetal risks. Shared decision-making, informed by the best available (though limited) evidence and the patient’s values, is essential. Transplant centers should develop structured protocols for preconception counseling that explicitly address the lack of belatacept safety data. The principle of informed consent becomes especially critical when continuing or initiating a medication with unproven fetal safety in a healthy or potentially viable pregnancy.

### 4.3. Clinical Implications

Rather than endorsing a specific management strategy, this review maps the options and considerations relevant to clinicians, because the available evidence—21 pregnancies from three uncontrolled reports—cannot support directive recommendations regarding continuation, conversion, or initiation of belatacept. For a recipient who conceives while stable on belatacept, the reported experience neither establishes fetal safety nor demonstrates that a switch to another regimen is required; any decision to continue, and the intensity of maternal–fetal monitoring (for example, blood-pressure surveillance, quantification of proteinuria, and serial fetal-growth assessment), should be individualized. For women planning pregnancy who require withdrawal of mycophenolate, belatacept is only one of several pregnancy-compatible strategies; alternatives such as azathioprine and calcineurin inhibitor-based regimens, together with baseline immunologic risk, prior rejection, graft function, Epstein–Barr virus serostatus, and transplant-center expertise, must all be weighed, and belatacept should not be presented as a generally preferred conversion target. De novo initiation of belatacept during pregnancy is not supported by the available data. All such decisions require individualized, multidisciplinary assessment involving transplant physicians, maternal–fetal-medicine specialists, and the patient, and should explicitly acknowledge that the certainty of the underlying evidence is very low.

Breastfeeding outcomes remain poorly characterized. The available reports did not identify adverse infant effects associated with breastfeeding during maternal belatacept therapy [12,14]. Given that belatacept is a large fusion protein with limited expected oral bioavailability, substantial systemic infant absorption appears unlikely. Nevertheless, formal pharmacokinetic and long-term pediatric safety studies are currently lacking.

### 4.4. Research Gaps and Priorities

Major gaps remain in the current understanding of belatacept exposure during pregnancy, underscoring the need for more rigorous and collaborative research efforts. Existing evidence regarding maternal safety is restricted almost entirely to isolated case reports and a single small case series, highlighting the urgent need for prospective multicenter observational studies with standardized maternal outcome reporting. Similarly, available fetal safety data remain insufficient to reliably assess uncommon congenital anomalies because the total number of exposed pregnancies is extremely small. Establishment of international pregnancy registries dedicated to biologic immunosuppressive agents would therefore be essential to improve statistical power and outcome surveillance.

Long-term pediatric outcomes represent another major unresolved area. At present, there are no meaningful data evaluating neurodevelopmental, immunologic, cognitive, or growth-related outcomes following in utero belatacept exposure. Longitudinal pediatric follow-up studies are needed to determine whether subtle delayed effects emerge later in childhood. Comparative effectiveness also remains poorly defined because no controlled studies have directly compared belatacept-based regimens with tacrolimus- or cyclosporine-based immunosuppression during pregnancy. Carefully designed comparative cohort studies would help clarify whether maternal, fetal, or graft outcomes differ meaningfully between these strategies.

Important uncertainties also persist regarding breastfeeding and placental pharmacology. No pharmacokinetic studies have evaluated belatacept concentrations in breast milk or infant serum, making maternal milk monitoring and infant exposure studies an important future priority. Likewise, human placental transfer data remain limited, and further mechanistic and pharmacokinetic placental studies are required to better characterize fetal exposure patterns throughout gestation. In addition, the implications of in utero exposure to a biologic immunosuppressant for the timing and safety of neonatal vaccination—particularly live vaccines—are unknown; this represents an evidence gap warranting careful, individualized consideration rather than a specific recommendation.

Interpretation of the currently available literature is further complicated by substantial confounding by indication, since belatacept has largely been reserved for recipients with complex transplant histories, calcineurin inhibitor toxicity, or difficult clinical courses. Future registry analyses incorporating risk adjustment and stratified outcome assessment will therefore be critical. Finally, the generalizability of current evidence remains limited because all reported cases originated from highly specialized transplant centers within the United States. International collaborative datasets involving broader and more diverse transplant populations are needed before definitive conclusions regarding safety and applicability can be established.

### 4.5. Limitations

This review has several important limitations that temper the strength of its conclusions.

First, the evidence base is exceptionally small, comprising only 21 pregnancies across three studies (one case series and two case reports). This sample size is inadequate to detect rare congenital anomalies or provide statistical reassurance. Second, there is no comparator group receiving standard calcineurin inhibitor-based therapy, so any claim of relative safety or superiority remains speculative. Third, publication bias is likely, as positive or uneventful outcomes are more often reported than negative ones. Fourth, follow-up duration was short (mostly ≤1 year postpartum), precluding assessment of long-term maternal graft survival or pediatric neurodevelopmental outcomes. Fifth, all cases originated from US transplant centers, limiting generalizability to other healthcare systems or lower-volume programs. Sixth, confounding by indication is substantial: belatacept was used predominantly in patients with prior calcineurin inhibitor toxicity or rejection, making it impossible to isolate drug effects from patient selection. Seventh, no trimester-specific exposure data were reported. Eighth, the retrospective design of the source studies introduces information bias. Ninth, the search was restricted to peer-reviewed publications; grey-literature sources—including transplant and pharmacovigilance registries, regulatory adverse-event databases, conference abstracts, and dissertations—were not systematically searched, which may have excluded relevant unpublished exposures and further contributed to publication bias, although it improved the consistency and feasibility of critical appraisal. Tenth, the review protocol was registered retrospectively, which reduces methodological transparency. Taken together, these factors mean that the overall certainty of the evidence is very low.

Despite these limitations, the present review provides the first structured synthesis of published pregnancy outcomes associated with belatacept exposure across solid organ transplantation. The findings support the need for international collaborative registries specifically designed to evaluate pregnancy after exposure to newer biologic immunosuppressive agents. Future research should include standardized maternal follow-up, detailed fetal imaging data, long-term pediatric assessment, placental pharmacokinetic studies, and comparative analyses against contemporary calcineurin inhibitor-based protocols.

From a clinical perspective, the currently available evidence does not justify routine initiation of belatacept during pregnancy and is insufficient to support any specific management strategy. Where continuation is considered in a recipient already stable on belatacept—particularly when alternative immunosuppressive strategies carry substantial maternal or graft-related risk—such decisions must be individualized and made through multidisciplinary assessment involving transplant physicians, maternal–fetal-medicine specialists, neonatologists, and the patient, recognizing that the certainty of the supporting evidence is very low.

## 5. Conclusions

This scoping review, conducted according to PRISMA-ScR guidelines, synthesized the available evidence on belatacept exposure during pregnancy in solid organ transplant recipients. Three studies (one case series and two case reports) reported 21 pregnancies among 15 recipients, mostly kidney transplant recipients, with several recipients contributing more than one pregnancy. Sixteen of the 21 pregnancies resulted in live birth and five ended in miscarriage, at least four of the latter occurring with periconception mycophenolate exposure. No congenital anomalies were reported among live-born infants, and stable allograft function was reported in 14 of 19 pregnancies with follow-up data. Maternal complications—particularly preeclampsia (8 of 16 in the case series), gestational diabetes, and infections—and low birth weight (<2500 g in all live births, largely reflecting prematurity) were common. The published cases have not identified congenital malformations to date, but the number of documented exposures is far too small to characterize teratogenic risk; the evidence base is noncomparative, derived from low-level observational data, and of very low certainty, and outcomes cannot be assumed equivalent to those of conventional immunosuppression. Belatacept therefore cannot be recommended for routine use during pregnancy, and clinical decisions must remain individualized. Preconception counseling, Epstein–Barr virus screening, close maternal–fetal monitoring, large prospective registries, and long-term pediatric follow-up are needed to establish safety and guide practice.

## Figures and Tables

**Table 1:** Eligibility criteria for study selection.

Category	Inclusion Criteria	Exclusion Criteria
Language	Studies published in or translated into English	Non-English studies without available translation
Timeframe	January 2015 to March 2025	Studies published before 2015
Study design	Original studies reporting clinical pregnancy outcomes, including case reports, case series, observational studies, cohort studies, and randomized trials	Reviews, editorials, conference abstracts without adequate clinical detail, protocols, animal studies, and grey literature
Population	Solid organ transplant recipients receiving belatacept-based maintenance immunosuppression during planned or unplanned pregnancy	Non-pregnant transplant recipients or recipients not exposed to belatacept
Organ type	Kidney, liver, heart, lung, or combined solid organ transplantation	Non-solid organ transplantation
Outcomes	Studies reporting maternal, fetal, neonatal, or allograft-related pregnancy outcomes	Studies lacking pregnancy-specific clinical outcomes

**Table 2:** Study and recipient characteristics.

Author (Year)	Study Design	Location	Recipients (n)	Pregnancies (n)	Organ	Time from Transplant to Pregnancy	Recipient Age	Pre-Pregnancy Graft/ Rejection History	Donor Type	Planned Pregnancy
Coscia et al. (2023) [12]	Case series	Georgia, USA	12	16	Kidney	Median 4 years (range 2–8)	Median 35.5 (31.5–37)	Prior rejection in 7/16	Living donor in 8/12	10/16
Combs et al. (2018) [13]—Patient A	Case report	Michigan, USA	1	3	Kidney	1.25, 2.5, and 3 years	28 years	Two acute rejections post-transplant; additional rejection after miscarriage	Living donor	All unplanned
Combs et al. (2018) [13]—Patient B	Case report	Michigan, USA	1	1	Kidney	4 years	36 years	Calcineurin inhibitor toxicity 1 year after transplantation	Living donor	Planned
Klintmalm et al. (2020) [14]	Case report	Texas, USA	1	1	Liver	5.5 years after second transplant	Not reported	Chronic antibody- mediated rejection before conversion to belatacept	First living donor, second deceased	Unplanned

**Table 3:** Concomitant immunosuppression at the time of conception or during pregnancy.

Study	Concomitant Immunosuppressive Agents	Mycophenolate Discontinued Before Conception?	Notes
Coscia et al. (2023) [12]	Mycophenolate (in some), steroids (variable), tacrolimus (in some)	Yes, in 11 of 16 pregnancies	Most patients transitioned off MMF before planned pregnancy
Combs et al. (2018) [13]—Patient A	Mycophenolate, prednisone	No—unplanned pregnancies; stopped after confirmation	Three pregnancies; two miscarriages occurred on MMF
Combs et al. (2018) [13]—Patient B	Prednisone, belatacept alone (no MMF)	Yes—stopped before conception	Stable graft function; preeclampsia developed
Klintmalm et al. (2020) [14]	Mycophenolate (discontinued several weeks before conception), then belatacept monotherapy	Yes—discontinued several weeks before conception	Liver recipient; no MMF exposure during pregnancy

Abbreviations: MMF, mycophenolate mofetil.

**Table 4:** Maternal, fetal, and allograft outcomes following belatacept exposure during pregnancy.

Author (Year)	Pregnancy Outcome	Mycophenolate Discontinued Before Conception	Gestational Age at Delivery	Follow-Up Duration	Maternal Outcomes	Neonatal/Fetal Outcomes	Breastfeeding	Certainty of Evidence (GRADE)
Coscia et al. (2023) [12]	13 live births; 3 miscarriages	Yes in 11/16 pregnancies	Median 37 weeks (34.8–37.9)	Median 47.75 weeks in 12 pregnancies; 4 lost to follow-up	Preeclampsia 8/16; COVID-19 2/16; CMV reactivation 1/16; GDM 1/16; worsening proteinuria/AKI 1/16	No congenital anomalies; low birth weight in all (13/13); NICU observation in 1 infant	7 mothers breastfed	Very low
Combs et al. (2018) [13]—Patient A	1 live birth; 2 miscarriages (8, 11 weeks)	No (conceived on MMF; stopped after confirmation)	Live birth at 38 weeks	6 months after third pregnancy	Acute cellular and antibody-mediated rejection after miscarriage; stable renal function until 3 months postpartum; graft loss at 6 months due to noncompliance	Healthy infant without congenital anomalies; low birth weight	Not reported	Very low
Combs et al. (2018) [13]—Patient B	1 live birth	Yes	37 weeks	14 months	Preeclampsia; stable graft function without renal complications	Healthy neonate without congenital anomalies; low birth weight	Not reported	Very low
Klintmalm et al. (2020) [14]	1 live birth	Yes (discontinued several weeks before conception)	35 weeks + 6 days	12 months	Stable graft function; cesarean delivery without major maternal complications	Healthy infant without congenital anomalies; low birth weight	Breastfed for 12 months without reported complications	Very low

Abbreviations: AKI, acute kidney injury; CMV, cytomegalovirus; COVID-19, coronavirus disease 2019; GDM, gestational diabetes mellitus; MMF, mycophenolate mofetil; NICU, neonatal intensive care unit.

**Table 5:** Comparative pregnancy considerations among common immunosuppressive agents used in solid organ transplantation.

Agent	Major Teratogenic Risk	Nephrotoxicity	Maternal Hypertension Risk	Pregnancy Experience	Key Pregnancy Concerns
Mycophenolate mofetil	High	No direct nephrotoxicity	Indirect	Contraindicated during pregnancy	Miscarriage, craniofacial and cardiac malformations
Tacrolimus	No major teratogenic signal	Yes	Moderate to high	Extensive clinical experience	Hypertension, diabetes, nephrotoxicity, prematurity
Cyclosporine	No major teratogenic signal	Yes	Moderate to high	Extensive clinical experience	Hypertension, nephrotoxicity, fetal growth restriction
Azathioprine	Low	Minimal	Low	Widely used during pregnancy	Bone marrow suppression at high doses
mTOR inhibitors	Uncertain; limited human data	Minimal	Variable	Limited pregnancy experience	Possible impaired fetal growth and wound healing concerns
Belatacept	No overt teratogenic signal reported to date	Minimal	Insufficient data	Very limited human pregnancy data	Insufficient safety evidence; lack of long-term pediatric data; EBV-related PTLD concern

Abbreviations: EBV, Epstein–Barr virus; PTLD, post-transplant lymphoproliferative disorder.

## Data Availability

Not applicable. This article is a scoping review of published literature; no new datasets were generated or analyzed for this study. All data discussed are available within the cited primary publications.

## Supplementary Materials

The following supporting information can be downloaded at: https://www.mdpi.com/article/10.3390/healthcare14162561/s1, Table S1. PRISMA Extension for Scoping Reviews (PRISMA-ScR) Checklist.

## Abbreviations

AKI, acute kidney injury; CMV, cytomegalovirus; COVID-19, coronavirus disease 2019; EBV, Epstein–Barr virus; FDA, Food and Drug Administration; GDM, gestational diabetes mellitus; MMF, mycophenolate mofetil; NICU, neonatal intensive care unit; PLLR, Pregnancy and Lactation Labeling Rule; PRISMA-ScR, Preferred Reporting Items for Systematic Reviews and Meta-Analyses Extension for Scoping Reviews; PTLD, post-transplant lymphoproliferative disorder.

## Appendix A

**Table A1:** Patient-level (per-pregnancy) summary of belatacept exposure, mycophenolate status, pregnancy planning, and maternal, neonatal, and graft outcomes across the included reports. Individual-level data for the Coscia et al. case series were not published and are therefore presented in aggregate. LBW, low birth weight (<2500 g); MMF, mycophenolate mofetil; NR, not reported; GA, gestational age; f/u, follow-up.

Recipient/Pregnancy	Study	Organ	Planned?	MMF at Conception	GA at Delivery	Birth Weight	Pregnancy, Maternal & Graft Outcome
Case series: 12 recipients/16 pregnancies	Coscia 2023 [12]	Kidney	Planned in 10/16	MMF continued at conception in 2 of 3 miscarriages; withdrawn before conception in 11/16	Median 37 wk (34.8–37.9)	LBW in all live births; individual values NR	13 live births, 3 miscarriages (2 with periconception MMF); preeclampsia 8/16; no rejection; graft function stable where reported; 4 lost to f/u
Recipient 13—Pregnancy 1	Combs 2018 [13] (Patient A)	Kidney	Unplanned	Yes (on MMF)	Miscarriage at 8 wk	–	First-trimester miscarriage on MMF
Recipient 13—Pregnancy 2	Combs 2018 [13] (Patient A)	Kidney	Unplanned	Yes (on MMF)	Miscarriage at 11 wk	–	First-trimester miscarriage on MMF; acute cellular + antibody-mediated rejection after miscarriage
Recipient 13—Pregnancy 3	Combs 2018 [13] (Patient A)	Kidney	Unplanned	Yes at conception; stopped after confirmation	Live birth at 38 wk	LBW; value NR	Live birth; graft loss 6 months postpartum (nonadherence to belatacept)
Recipient 14—Pregnancy 1	Combs 2018 [13] (Patient B)	Kidney	Planned	No (belatacept + prednisone; no MMF)	Live birth at 37 wk	LBW; value NR	Preeclampsia; stable graft function; 14-month f/u
Recipient 15—Pregnancy 1	Klintmalm 2020 [14]	Liver	Unplanned; MMF stopped pre-conception	No (discontinued several weeks before conception)	Live birth at 35 wk + 6 d	LBW; value NR	No maternal complications; stable graft; breastfed 12 months; 12-month f/u
