# Supplementary material for: Pregnancy Outcomes After Belatacept Exposure in Solid Organ Transplant Recipients: A Scoping Review
Source: Healthcare (Basel). 2026 Aug 16;14(16):2561. doi: 10.3390/healthcare14162561 (PMC13512030; doi:10.3390/healthcare14162561)
Supplement: Supplementary file 1 [file healthcare-14-02561-s001.zip › healthcare-4417964-supplementary.pdf]

Supplementary Table S1. PRISMA Extension for Scoping Reviews (PRISMA-ScR) Checklist

Tricco AC, Lillie E, Zarin W, et al. PRISMA Extension for Scoping Reviews (PRISMA-ScR): Checklist and explanation. Ann Intern Med. 2018;169:467–473.

Section	#	Checklist Item	Reported?	Location in Manuscript
**TITLE**				
TITLE	1	Identify the report as a scoping review.	Yes	Title page
**ABSTRACT**				
ABSTRACT	2	Provide a structured summary that includes, as applicable: background, objectives, eligibility criteria, sources of evidence, charting methods, results, and conclusions that relate to the review questions and objectives.	Yes	Abstract
**BACKGROUND**				
BACKGROUND	3	Describe the rationale for the review in the context of what is already known. Explain why the review questions/objectives lend themselves to a scoping review approach.	Yes	Section 1 (Introduction)
BACKGROUND	4	Provide an explicit statement of the questions and objectives being addressed with reference to their key elements (e.g., population or participants, concept, and context [PCC]).	Yes	Section 1 (Introduction)
**METHODS**				
METHODS	5	If a protocol exists, indicate whether it can be accessed (e.g., Web address); and if available, provide registration information including registration number.	Yes	Section 2 (Methods, para. 1). Retrospectively registered in OSF.
METHODS	6	Specify characteristics of the sources of evidence used as eligibility criteria (e.g., years considered, language, and publication status), and provide a rationale.	Yes	Section 2 (Methods); Table 1
METHODS	7	Name all databases searched or consulted (at minimum) and the date of the most recent search for each database.	Yes	Section 2 (Methods, para. 2)
METHODS	8	Present the full search strategy for at least one database including any limits used, such that it could be repeated.	Yes	Section 2 (Methods, para. 2 — full PubMed

				search string provided)
METHODS	9	State the process for selecting sources of evidence (i.e., screening and eligibility) included in the scoping review.	Yes	Section 2 (Methods, para. 4)
METHODS	10	Describe the methods of charting data from the included sources of evidence (e.g., calibrated forms or forms that have been piloted by the team before their use, and whether data charting was done independently or in duplicate).	Yes	Section 2 (Methods, para. 5)
METHODS	11	List and define all variables for which data were sought and any assumptions and simplifications made.	Yes	Section 2 (Methods, para. 5)
METHODS	12	If done, provide a rationale for conducting a critical appraisal of included sources of evidence; describe the methods used and how this information was used in any data synthesis.	Yes	Section 2 (Methods, para. 6)
METHODS	13	Describe the methods of handling and summarizing the data that were charted.	Yes	Section 2 (Methods, para. 5–6)
**RESULTS**				
RESULTS	14	Give numbers of sources of evidence screened, assessed for eligibility, and included in the review, with reasons for exclusions at each stage, ideally using a flow diagram.	Yes	Section 3.1; Figure 1 (PRISMA flow diagram)
RESULTS	15	For each source of evidence, present characteristics for which data were charted and provide the citations.	Yes	Section 3.2; Tables 2–3
RESULTS	16	If done, present data on critical appraisal of included sources of evidence.	Yes	Section 3.2 (Methods quality assessment)
RESULTS	17	For each included source of evidence, present the relevant data that have been charted that relate to the review questions and objectives.	Yes	Sections 3.2–3.4; Tables 2–4
RESULTS	18	Summarize and/or present the charting results as they relate to the review questions and objectives.	Yes	Section 3 (Results); narrative synthesis
**DISCUSSION**				

DISCUSSION	19	Summarize the main results (including an overview of concepts, themes, and types of evidence available), explain how they relate to the review questions and objectives, consider the possible implications of the findings, and identify knowledge gaps.	Yes	Section 4 (Discussion)
DISCUSSION	20	Discuss the limitations of the scoping review process.	Yes	Section 4.5 (Limitations)
DISCUSSION	21	Provide a general interpretation of the results with respect to the review questions and objectives, as well as potential implications and a statement about future research.	Yes	Section 5 (Conclusions); Section 4.4
**FUNDING**				
FUNDING	22	Describe sources of funding for the included sources of evidence, as well as sources of funding for the scoping review. Describe the role of the funder(s) of the scoping review.	Yes	Funding statement

OSF = Open Science Framework. * Registration DOI to be inserted after retrospective registration.
